# Tracking circulating PD-L1-positive cells to monitor the outcome of patients with gastric cancer receiving anti-HER2 plus anti-PD-1 therapy

**DOI:** 10.1007/s13577-023-00990-8

**Published:** 2023-10-27

**Authors:** Xiaoyi Chong, Yanyan Li, Jialin Lu, Xujiao Feng, Yilin Li, Xiaotian Zhang

**Affiliations:** Key Laboratory of Carcinogenesis and Translational Research (Ministry of Education/Beijing), Department of Gastrointestinal Oncology, Peking University Cancer Hospital and Institute, Fu-Cheng Road 52, Hai-Dian District, Beijing, 100142 China

**Keywords:** HER2, PD-L1, Gastric cancer, CTCs, CECs, Karyotype shifting, Liquid biopsy, SE-iFISH

## Abstract

Dual blockade of HER2 and PD-1/PD-L1 is the most promising regimen for HER2-positive patients with gastric cancer (GC); PD-L1 combined positive score, rather than HER2 status, indicates potential benefit. Circulating tumor cells (CTCs) and circulating endothelial cells (CECs) derived from the tumor microenvironment provide platforms for the dynamic evaluation of PD-L1 expression. Whether PD-L1 positive CTCs/CECs (PD-L1^+^CTCs/CECs) can serve as biomarkers for evaluating the efficacy of combination therapy remains unknown. Therefore, this study investigated PD-L1 expression and heterogeneous karyotypic features of CTCs/CECs and their involvement in the clinical response to treatment in 72 patients with advanced GC by applying a pre-established surface molecule-independent subtraction enrichment (SE)-iFISH strategy. In the captured PD-L1 positive cells, there were 42.80% and 57.20% of CTCs and CECs, respectively. PD-L1^+^ CTCs were pre-therapeutically detected in 0% (0/11) of HER2-negative patients and 14.75% (9/61) of HER2-positive patients. The presence of baseline PD-L1^+^CTCs was relevant to inferior prognosis (mPFS: 14.40 months vs 5.00 months, *P* = 0.065); post-treatment PD-L1^+^ CECs were associated with longer irPFS (immunotherapeutic-related PFS) (mPFS: 15.57 months vs 6.73 months, *P* = 0.053). Further dynamic karyotype-based profiling of PD-L1^+^ CTCs/CECs indicated that multiploidy and triploidy were the dominant subtypes of baseline PD-L1^+^ CTCs, and that triploidy was specifically associated with therapeutic resistance. Intratherapeutically detected multiploid PD-L1^+^ CECs demonstrated a superior clinical response; triploidy and tetraploidy contributed to acquired resistance. The karyotypic features of PD-L1^+^CTCs/CECs should be dynamically profiled in patients with GC treated with anti-HER2 plus anti-PD-1 therapy. Triploid-PD-L1^+^ CTCs and multiploid-PD-L1^+^ CECs are potential indicators of therapeutic response.

## Introduction

Gastric cancer (GC) is the most common worldwide malignancy with high morbidity and mortality rates [1]. Human epidermal growth factor receptor-2 (HER2) was known as a member of the tyrosine kinase receptor family. Overexpression of HER2 leads to the formation of its various dimers, activation of MAPK, PI3K, JAK–STAT3, and PKC pathways evoking malignant development and tumor progression. Approximately 13% of advanced GC patients are histopathologically positive for human epidermal growth factor receptor-2 (HER2) [2]. An open label, international, phase 3, randomized-controlled trial (ToGA) enrolled 594 HER2-positive gastric or gastroesophageal junction adenocarcinoma patients indicated that compared with chemotherapy alone, trastuzumab (a monoclonal antibody against HER2) plus chemotherapy significantly improved patients survival (mOS: 11.1 moths vs 13.8 moths, p = 0.0046). Since then, trastuzumab plus chemotherapy became the standard 1st-line therapy for HER2-positive gastric or gastroesophageal junction adenocarcinoma patients[3]. Trastuzumab combined with chemotherapy remains the mainstay treatment for HER2-positive GC. Previous studies have shown that the antibody-dependent cell-mediated cytotoxicity (ADCC) of trastuzumab increases the expression of PD-L1 on T and tumor cells [4]. A recent phase III Keynote-811 study indicated that trastuzumab combined with pembrolizumab and chemotherapy markedly revolutionized the treatment of unresectable or metastatic HER2-positive GC or gastroesophageal junction adenocarcinoma [5]. A 22.7% improvement in the objective response rate (ORR) of first-line treatment has been reported, indicating that anti-HER2 and anti-PD-1 therapy is the most promising regimen for patients with HER2-positive GC. A few studies have explored the potential biomarkers of anti-HER2 plus anti-PD-1 therapy. Keynote-811 adjoint analysis demonstrated that instead of HER2 status, the PD-L1 combined positive score (CPS) ≥ 1 and microsatellite instability (MSI)-high status were associated with a high likelihood of response to treatment.

Although multiple studies with immune checkpoint inhibitors have shown similar responses in patients regardless of PD-L1 status, the expression of PD-L1 is generally considered to be a factor representing favorable clinical outcomes: Checkmate 649 additional analysis in patients with GC with CPS ≥ 5 revealed more durable responses [6]. Retrospective analysis of Keynote-059 [7], Keynote-061 [8], and Keynote-062 [8] suggested improvements toward more favorable clinical outcomes with pembrolizumab across lines of therapy in patients with CPS ≥ 10 (Gastric/Gastroesophageal junction, G/GEJ) cancer. The association between HER2 and PD-L1 expression remains unclear, although higher expression of PD-L1 has been found in trastuzumab-resistant HER2-positive cells [9]. Therefore, it is necessary to continuously evaluate PD-L1 expression to screen potential beneficiaries and monitor the development of resistance during anti-HER2 plus anti-PD-1 therapy. Nevertheless, the heterogeneous expression between primary and metastatic tumors and dynamic changes during treatment severely impair the predictive value of PD-L1 immunohistochemistry (IHC).

Circulating tumor cells (CTCs) derived from primary or metastatic tumor sites provide complementary information regarding metastasis and therapeutic responses [10, 11]. The presence of PD-L1^+^ CTCs in peripheral blood has been shown to have immunotherapeutic prognostic relevance in many studies. Peripheral blood tumor-associated circulating endothelial cells (CECs), which carry characteristics of tumors and stem cells participating in tumor angiogenesis and dysfunctional angiogenic vasculatures, further promote distant metastasis and progression [12–14]. PD-L1^+^ CECs exhibit resistance to checkpoint blockade immunotherapy and have been reported in non-small cell lung cancer [15]. Aneuploidy is also an essential feature of CTCs and tumor-associated CECs [16, 17]. Previous studies have indicated that aneuploidy can affect the transcription of multiple genes and tumor heterogeneity [18]. Various aneuploid CTCs/CECs that escape from tumor sites participate in tumor resistance, recurrence, and metastasis. Heterogeneous-aneuploid CTCs/CECs harbor distinct genetic signatures, thereby participating in therapeutic responses through diverse pathways. Whether different PD-L1-positive, aneuploid CTC/CECs can serve as biomarkers to evaluate anti-HER2 plus anti-PD-1 efficacy remains unclear.

Based on a pre-established surface molecule-independent subtraction enrichment (SE)-iFISH strategy, the presence of PD-L1^+^ CTCs/CECs and their impact on therapy were explored using longitudinal analyses in patients receiving anti-HER2 plus anti-PD-1 therapy. In particular, we investigated the different aneuploid distribution in PD-L1^+^ CTC/CECs and their effects on therapeutic resistance.

## Materials and methods

### Patient selection and information

A total of 72 patients with advanced GC were enrolled at Peking University Cancer Hospital between June 2020 and June 2022. All patients were ≥ 18 years of age and were diagnosed with unresectable or metastatic GC [including both gastroesophageal junction (GEJ) and non-GEJ types]. Patients with an Eastern Cooperative Oncology Group performance status of 0/1, measurable or non-measurable but evaluable lesions, and adequate organ function were eligible for this study. The enrolled patients received first- or second-line anti-HER2 plus anti-PD-1 and cisplatin or paclitaxel chemotherapy.

The patients’ clinical responses were evaluated every 6 weeks using computed tomography (CT) based on the ir-RECIST criteria. Therapy responses were categorized as complete response (CR), partial response (PR), stable disease (SD), or progressive disease (PD). Clinical responses never reaching complete response (CR), partial response (PR), or stable disease (SD) were defined as primary resistance, whereas those which achieved overall disease control (CR, PR, or SD) and finally developed PD were regarded as acquired resistance. HER2 positivity in tumor tissues was histopathologically indicated as either IHC 3 + or IHC 2 + with ERBB2 amplification. The PD-L1 combined positive score (CPS) is the proportion of PD-L1-positive cells among all tumor cells. Six milliliters (mL) of peripheral blood was periodically collected from recruited patients at baseline and post-treatment. Informed consent was obtained from all the patients. This study was approved by the Institutional Ethics Committee of Peking University Cancer Hospital and Institute (ID: 2020KT46) and was conducted in accordance with the Declaration of Helsinki.

### Enrichment and characterization of aneuploid PD-L1 positive CTC/CEC using SE-iFISH

Subtraction enrichment was performed according to the manufacturer’s instructions (Cytelligen) and previously reported protocols [19]. Briefly, 6 mL of peripheral blood was collected in a tube containing the ACD anticoagulant (Becton Dickinson). Samples were centrifuged to remove plasma, and the sedimented blood cells were resuspended in 3 mL of hCTC buffer and loaded onto a non-hematologic cell separation matrix. After centrifugation, white blood cells (WBCs) and tumor cells were collected and incubated with immunomagnetic beads to obtain anti-WBC monoclonal antibodies (mAbs). White blood cells (WBCs) were removed using a magnetic stand. The remaining solution was centrifuged and the sedimented cells were mixed and smeared on formatted CTC slides. The specimens were dried for subsequent iFISH analyses.

PD-L1/CD31-iFISH was performed according to the manufacturer’s protocol. Briefly, dried monolayers of cells on coated slides were hybridized with a Vysis chromosome 8 centromere probe (CEP8) Spectrum Orange (Vysis, Abbott Laboratories, Chicago, IL, USA) to identify various aneuploid cells. The slides were then incubated with Alexa Fluor (AF) 594-CD45 (ATCC, Manassas, VA, USA, Clone 9.4), AF488-PD-L1 (Dana-Farber Cancer Institute, Harvard Medical School, Boston, MA, USA, Clone 29E.2A3), and Cy5-CD31 (Abcam, Burlingame, CA, USA, Clone EP3095). After washing, the samples were mounted with mounting media containing DAPI (Vector Laboratories, Burlingame, CA, USA), and subsequently subjected to automated Metafer-i-FISH ® three-dimensional scanning and an image analyses system co-developed by Carl Zeiss (Oberkochen, Germany), MetaSystems (Altlussheim, Germany), and Cytelligen [20]. PD-L1^+^ CTCs/CECs feature as DAPI^+^CD45^−^PD-L1^+^CD31^−^/ DAPI^+^CD45^−^PD-L1^+^CD31^+^ with diploid and aneuploid Chr8 expression, respectively.

### Statistical analysis

All statistical analyses were performed using SPSS software (version 21.0; IBM Corp., Armonk, NY, USA) and GraphPad Prism 9.0. The correlation of PD-L1 positive cells’ numbers with CPS was analyzed using the Spearman Rank correlation test. Progression-free survival (PFS) was defined as the time from the initial treatment to the date when clinical disease progression was diagnosed or censored at the last follow-up. Overall survival (OS) was defined as the time from regimen administration to the date of death or censored at the last follow-up. Kaplan–Meier survival plots for PFS and OS were generated based on the number of diverse aneuploid PD-L1^±^ CTCs and CECs. Log-rank tests were used to compare the survival curves. All *P* values were two-sided, and a *P* value < 0.05 was considered statistically significant.

## Results

### Patients’ characteristics

This study prospectively enrolled 72 patients with gastric cancer who received treatment, including dual HER2 and PD1 blockade therapy. The clinicopathological characteristics of the 72 evaluable patients are summarized in Table 1. Pre-therapeutic HER2 status was evaluated, and 11 of 72 patients were diagnosed as HER2-negative (HER2 low expression), of which seven were IHC +  + with *ERBB2* non-amplification and four were IHC + with *ERBB2* non-amplification. The HER2-negative patients were all treated with RC48 plus anti-PD1 (ClinicalTrials.gov ID: NCT04280341) PD-L1^+^CTCs were detected in 0% (0/11) of HER2-negative patients and 14.75% (9/61) HER2-positive patients, while PD-L1^+^ CECs were detected in 27.27% (3/11) and 18.03% (11/61) of HER2-negative and HER2-positive patients, indicating a distinct distribution of PD-L1^+^ circulating cells in patients with different HER2 statuses. The clinical responses of 66 (91.70%) patients were assessable, of whom 49 (68.1%) and 17 (23.6%) received anti-HER2 plus anti-PD-1 as first- and later-line treatment regimens, respectively.

**Table 1:** Baseline characteristics of enrolled patients

Variable	No. of patients. (%)
Sex	
Male	58 (80.6%)
Female	14 (19.4%)
Histopathology	
Non-EGJ	45 (62.5%)
EGJ	27 (37.5%)
Lauren	
Intestinal	51 (70.8%)
Diffuse	4 (5.6%)
Mixed	11 (15.3%)
NA	6 (8.3%)
HER2 status	
Positive	61 (84.7%)
Negative	11(15.3%)
PD-L1 status	
CPS ≥ 1	19 (26.4%)
CPS < 1	28 (38.9%)
NA	25 (34.7%)
MMR status	
PMMR	69 (95.8%)
DMMR	0 (0%)
NA	3 (4.2%)
Therapeutic line	
Neoadjuvant/adjuvant	6 (8.3%)
First	49 (68.1%)
Later	17 (23.6%)
Treatment regimens	
Chemotherapy-free	43 (59.7%)
Chemotherapy	29 (40.3%)
Best response	
PR	43 (59.7%)
SD	12 (16.7%)
PD	11 (15.3%)
Non-relapse	1 (1.4%)
NA	5 (6.9%)
Median survival (months)	
PFS	7.03
OS	Undefined

### Detection of aneuploid CTCs and CECs expressing PD-L1 using SE-iFISH

**Fig. 1:**
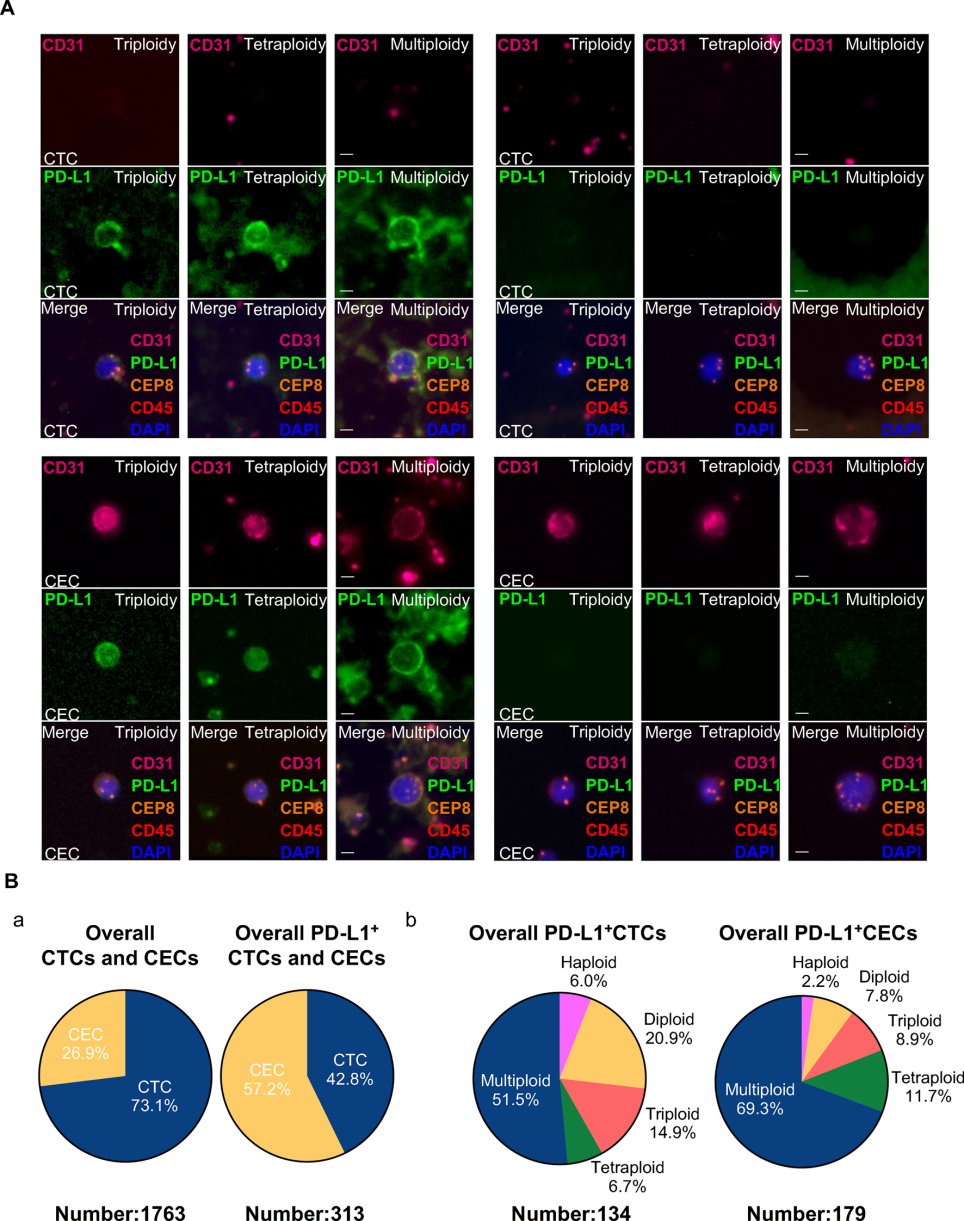
Detection of various subtypes of aneuploid CTCs and CECs expressing PD-L1 in G/GEJ cancer patients by SE-iFISH. **A** Characterization of CTCs and CECs in G/GEJ cancer patients by SE-iFISH. **B** PD-L1 positivity and karyotypic analysis CTCs and CECs detected in overall G/GEJ cancer patients. **a** Percentage of CTCs and CECs in total captured cells and PD-L1-positive cells. **b** Karyotypic distribution of PD-L1^+^ CTCs and CECs

Six-channel bi-marker-iFISH was developed and applied to evaluate PD-L1 expression and karyotypic features of aneuploid CTCs (CD31^−^/CD45^−^) and CECs (CD31^+^/CD45^−^) enriched by SE from patients’ pre- or post-therapeutic peripheral blood. PD-L1 was localized to the plasma membrane (Fig. 1A). Various aneuploid PD-L1 positive or negative CTCs or CECs are shown in Fig. 1A.

A total of 1763 cells was captured from baseline or intra-therapeutic samples, of which 17.75% (313/1763) were PD-L1 positive. Although CECs accounted for only 26.90% of the total cells, 57.20% identified PD-L1-positive cells were CECs, suggesting that PD-L1 positivity was substantially higher in CECs. Quantitative analysis of PD-L1 positive cells’ karyotypic subtypes’ composition revealed that multiploidy was the most common subtype in CTCs (51.50%) and CECs (69.30%).

### PD-L1 expression in CTCs/CECs/matched tissues

**Fig. 2:**
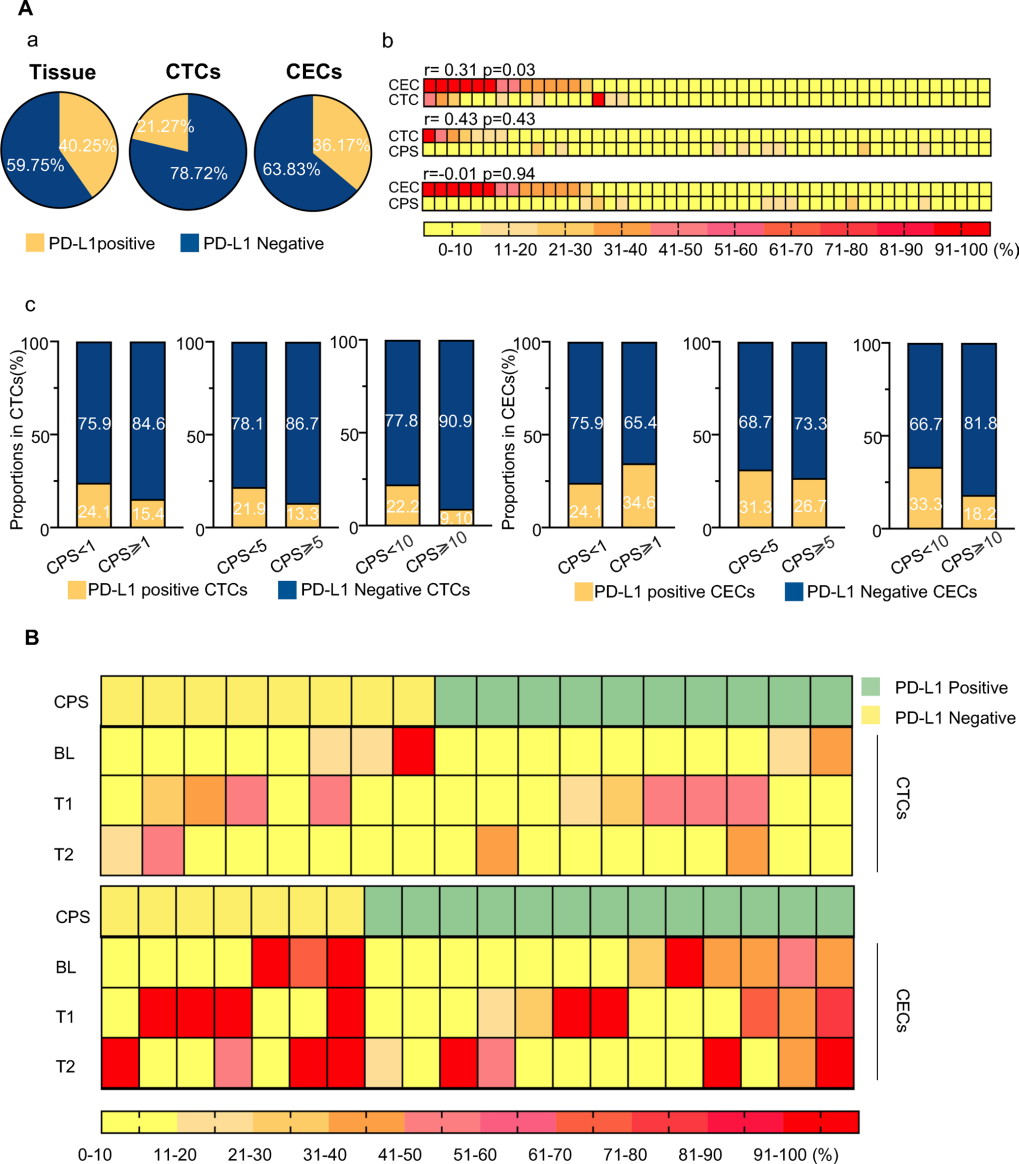
PD-L1 expression on CTC/CECs at baseline and the dynamic acquisition during therapy. **A** Comprehensive analysis of PD-L1 expression on primary tumor sites, CTCs, and CECs prior to therapy (**a**) PD-L1 positivity of primary tumor sites, CTCs, and CECs at baseline; (**b**) Spearman rank correlation analysis of CPS, PD-L1 positivity of CTCs and CECs; (**c**) differences CTCs and CECs PD-L1 positivity in distinct CPS (cut-off 1, 5, 10). **B** Dynamic variation of PD-L1-positive CTCs and CECs during therapy. T: time for collected samples

CTCs, CECs, and tumor tissues exhibited heterogeneous expression of PD-L1 in this study. Among the 47 patients with baseline tissue PD-L1 CPS and CTC/CEC PD-L1 expression results, 40.25% (19/47) were positive for tissues, and 21.27% (10/47) and 36.17% (17/47) of patients were PD-L1 positive on CTCs and CECs, respectively (Fig. 2A-a). PD-L1 expression in CTCs correlated positively with CEC positivity (*r* = 0.31, *P* = 0.03) (Fig. 2A-b). No correlation was observed between the presence of PD-L1^+^CTCs/PD-L1^+^CECs and the PD-L1 CPS. Accordingly, there was no difference in the proportion of patients with PD-L1^+^CTCs/PD-L1^+^CECs detected at baseline between PD-L1-positive or -negative cohorts regardless of the CPS cut-off value (Fig. 2A-c).

### Enumeration and positivity of PD-L1^+^CTCs/CECs’ correlation to therapeutic response

Among the 47 subjects, 20 participants were longitudinally monitored during therapy. The positivity of PD-L1^+^CTCs/CECs fluctuated during therapy in both PD-L1-positive or -negative cohorts (Fig. 2B). Patients with no PD-L1 expression in tissue were able to acquire the PD-L1^+^ CTCs/CECs phenotype during treatment, indicating that 87.50% (7/8) of PD-L1 negative patients, and 80% (8/10) of PD-L1-negative patients could capture PD-L1^+^ CTCs at different times after treatment initiation. Intriguingly, almost all patients possessed PD-L1^+^ CECs after therapy despite the diverse PD-L1 status (Fig. 2B), suggesting that PD-L1^+^ CTCs/CECs can be continuously formed during therapy.

**Fig. 3:**
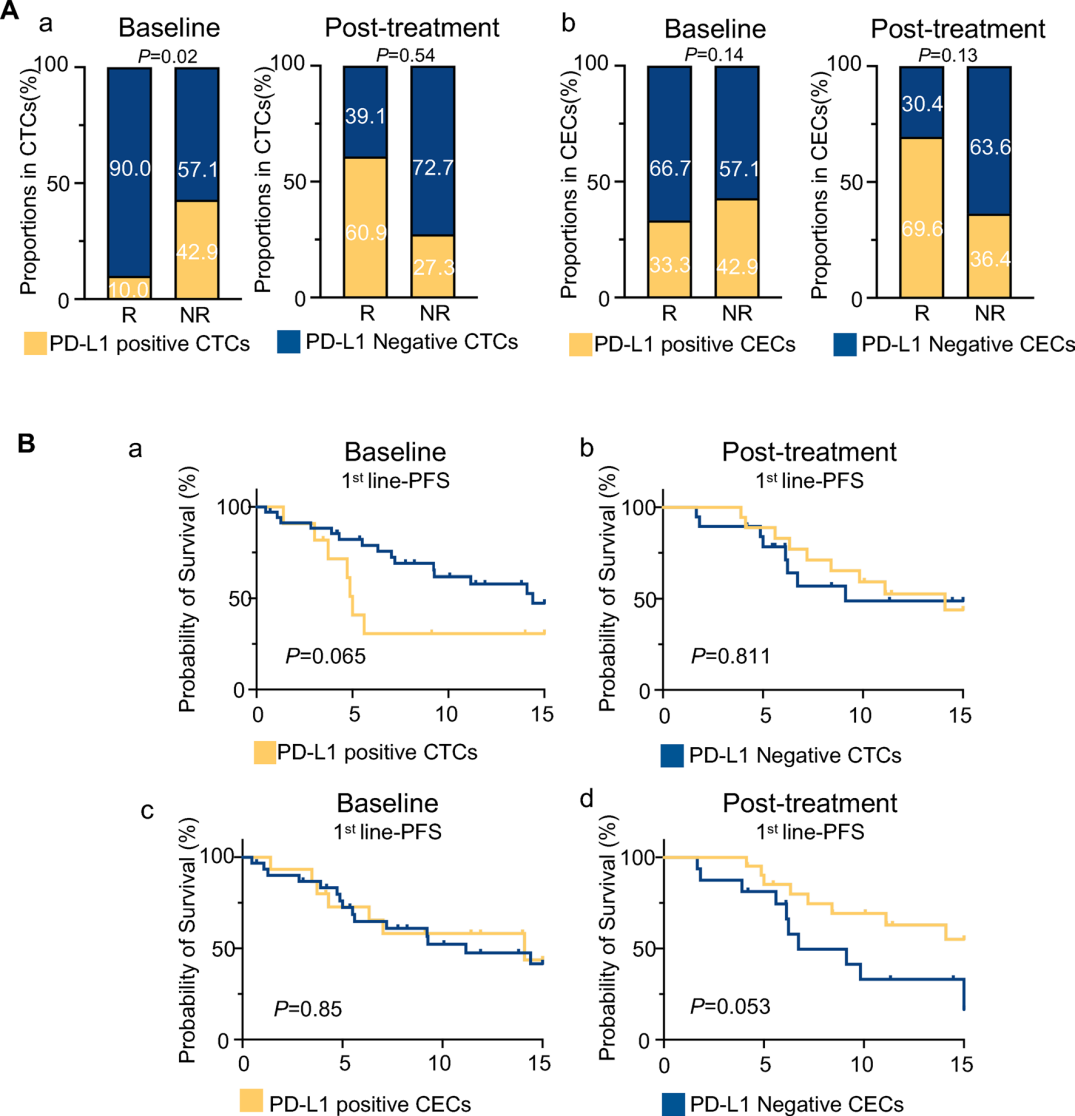
The associations of PD-L1 positive CTCs and CECs with therapeutic response and prognosis. **A** The histogram shows the distributions of PD-L1-positive CTCs and CECs proportion before and after treatment in R and NR, respectively. R, responders; NR, non-responders. **B** Kaplan–Meier curves of first-line PFS in relation to the pre- (**a**, **c**) post-treatment (**b**, **d**) proportion of PD-L1 positive CTCs (**a**, **b**) and PD-L1-positive CECs (**c**, **d**)

To explore the exact relationship between the pre- or post-treatment presence of PD-L1^+^ CTCs/CECs and therapeutic response, we analyzed 49 patients who received anti-HER2 plus anti-PD1 ± chemotherapy as first-line treatment. Five patients were excluded, because CTC/CEC images were not captured. Patients with a response of PR lasting for at least 6 months were regarded as responders (R), whereas those with a response of SD/PD or a PR duration shorter than 6 months were regarded as non-responders (NR). The distribution of pre- and post-treatment PD-L1^+^ CTCs/CECs in the R and NR groups was compared (Fig. 3A). The proportion of baseline PD-L1^+^ CTCs in the NR group was higher than that in the R group (42.90% vs. 10.00%, *P* = 0.02) (Fig. 3A-a). Although the *P* value did not reach significance, the trend indicated that pre-therapeutic PD-L1^+^CTCs were associated with inferior PFS in Kaplan–Meier analysis (mPFS: 14.40 months vs 5.00 months, *P* = 0.065). What’s more, the presence of intra-therapeutic PD-L1^+^CECs were found to be associated with superior PFS (mPFS: 15.57 m vs 6.73 m, *P* = 0.053) (Fig. 3B-d). Taken together, CTCs and CECs exerted different functions as biomarkers; pre-treatment PD-L1^+^CTCs and post-treatment PD-L1^+^CECs participated in the therapeutic response and were correlated with prognosis.

### Different aneuploid PD-L1^+^ CTCs/CECs exert distinct influences on therapeutic outcome

**Fig. 4:**
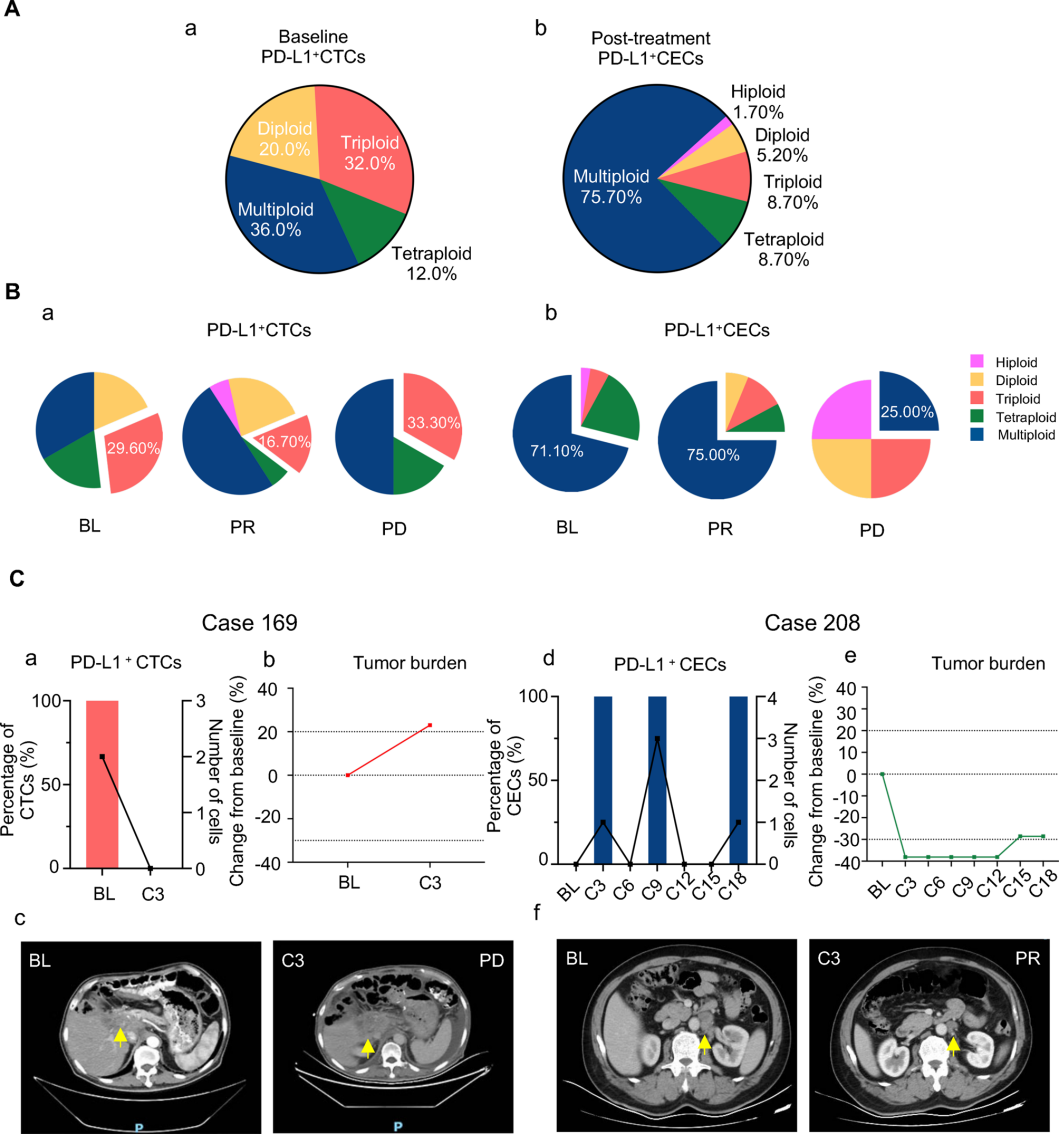
Karyotypic analysis of aneuploid PD-L1-positive CTCs and CTECs detected in pre- and post-treatment. **A** Karyotype distribution of baseline PD-L1^+^ CTCs (**a**) and post-treatment PD-L1^+ ^CECs (**b**). **B** Dynamic karyotypic shifting of PD-L1^+^CTCs (**a**) and PD-L1^+ ^CECs (**b**). **C** Individual case analysis of PD-L1^+^ aneuploid CTCs and CECs in patients subjected to the combination treatment. Case 169 and Case 208 PD-L1^+^ cells’ numbers’ positivity, karyotypic (**a**, **d**) tumor burden (**b**, **e**) changes along with the treatment and corresponding CT images (**c**, **f**) BL: baseline, *C* Cycle

Given the distinction of PD-L1^+^ CTCs/CECs in predicting clinical outcomes, we investigated the key aneuploidy in pre-treatment PD-L1^+^ CTCs and post-treatment CECs contributing to the therapeutic response further. A percentage of 36.00% and 32.00% baseline PD-L1^+^ CTCs were multiploid and triploid (Fig. 4A); 75.70% post-treatment PD-L1^+^ CECs were multiploid. The dynamic quantitative variations in different karyotypic PD-L1^+^ CTCs/CECs following treatment are also summarized. The percentage of triploid PD-L1^+^ CTCs decreased in the PR samples but increased in the PD samples (Fig. 4B-a). In contrast, the positivity of multiploid-PD-L1^+^ CECs surged in PR samples and dramatically declined in PD samples (Fig. 4B-b). Overall, baseline triploid-PD-L1^+^ CTCs were a specific subtype involved in therapeutic resistance and were correlated with poor prognosis, whereas intra-therapeutic multiploid-PD-L1^+^ CECs indicated a better clinical response. Two patients with homologous clinical features and contrasting therapeutic outcomes demonstrated the function of PD-L1^+^ CTCs and PD-L1^+^ CECs. Case 169: Case 169 was diagnosed with moderately differentiated gastric adenocarcinoma with liver metastasis (Fig. 4C-a-c). The patient’s Lauren histological type was intestinal, HER2 IHC +  + , with *ERBB2* amplification. Above all, Case 169 should be potential beneficiary of dual blockade of HER2 and PD-1. This patient was treated with first-line trastuzumab and pembrolizumab. Two triploid/6 mL PD-L1^+^CTCs were detected before therapy, and the patient developed PD after three treatment cycles. Case 208: the patient was diagnosed with moderately differentiated gastric adenocarcinoma with abdominal lymph-node metastasis (Fig. 4C-d-f). The patient’s Lauren histologic type was intestinal, HER2 IHC +  +  + . Treatment regimens were the same as those in case 169. Multiploid-PD-L1^+^ CECs were continuously detected during therapy, and the patient could benefit from the treatment until the end of follow-up.

**Fig. 5:**
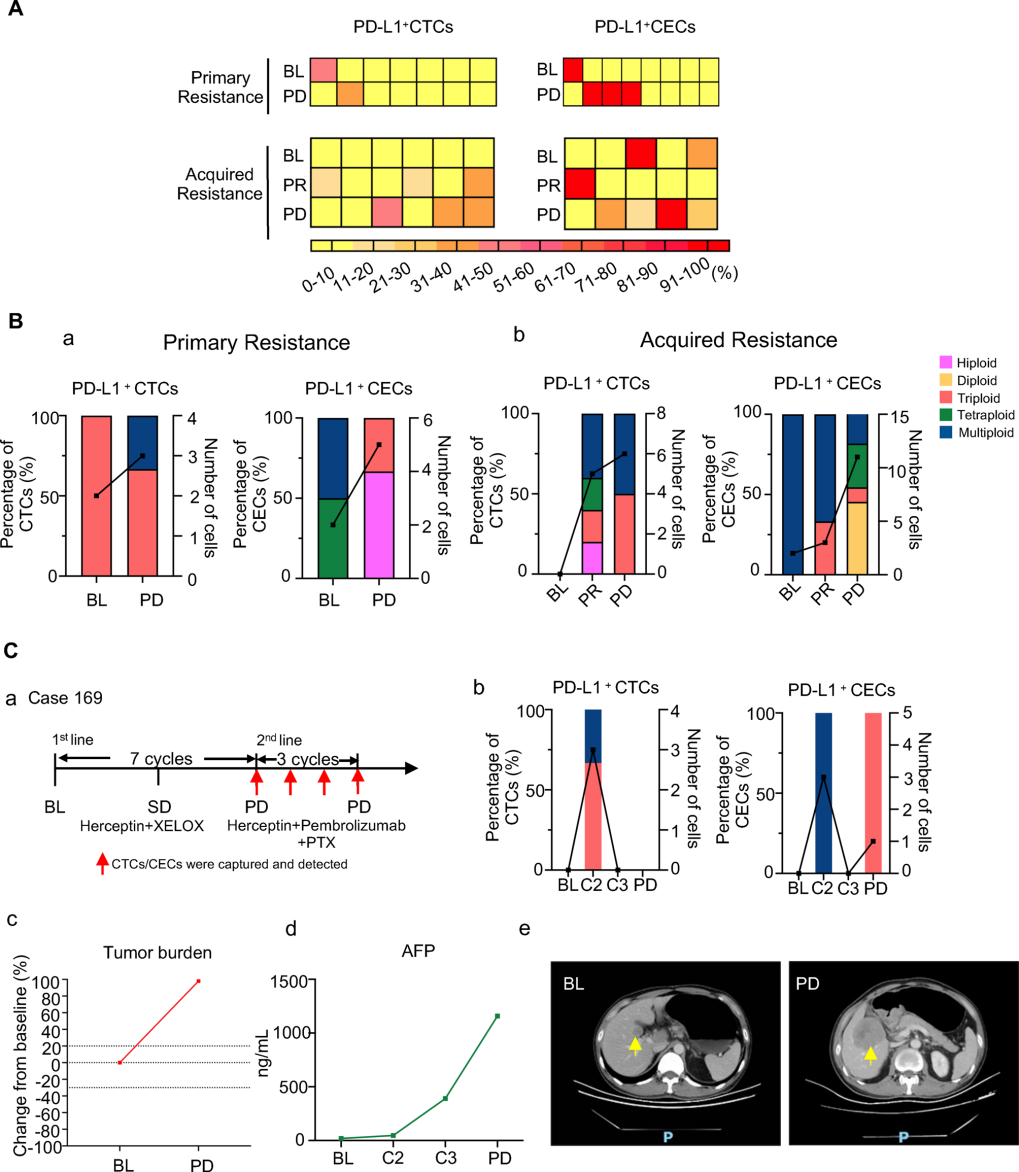
The associations of aneuploid PD-L1-positive CTCs/CECs with therapeutic resistance. **A** Dynamic changes of PD-L1 positivity of CTCs/CECs in primary resistant and acquired resistant patients. **B** PD-L1 + CTCs/CECs karyotypic changes in primary resistant (**a**) and acquired resistant (**b**) patients. **C** Representative case analysis of PD-L1 + aneuploid CTCs and CECs serving as disease progression indicators. Patients’ treatment pathway (**a**). PD-L1 + cells’ numbers’ positivity, karyotypic (**b**), tumor burden (**c**), and biomarker (**d**) changes along with the treatment and corresponding CT images (**e**)

To further explore the relationship between the presence of PD-L1^+^ CTCs/PD-L1^+^ CECs and primary and secondary therapeutic resistance, eight primary and six patients with acquired resistance with longitudinally assessable enumeration of CTCs/CECs were analyzed. A percentage of 14.29% (1/7) and 37.50% (3/8) of patients with primary resistance acquired PD-L1^+^ CTCs and PD-L1^+^ CECs, respectively, when therapeutic resistance developed (Fig. 5A). Fifty percent (3/6) and 80% (4/5) of the patients with acquired resistance had PD-L1^+^ CTCs and PD-L1^+^ CECs phenotypes, respectively, at the time of PD. These results suggest that PD-L1^+^ cell positivity may contribute to the development of primary and secondary resistance. Dynamic karyotypic distribution analysis demonstrated that PD-L1^+^ cells in PD displayed high heterogeneity. In patients with primary resistance, triploid-PD-L1^+^ CTCs were detected at baseline (Fig. 5B-a). In addition, the proportion of triploid-PD-L1^+^ CTCs increased substantially at the time of PD in patients with acquired resistance (Fig. 5B-b), showing that triploid-PD-L1^+^ CTCs participated in primary/secondary therapeutic resistance before and after treatment. Although the number of PD-L1^+^ CECs markedly increased when the disease reached PD, the percentage of multiploid-PD-L1^+^CECs diminished at PD, while multiple other aneuploid cells appeared. Given the finding that intra-therapeutic multiploidy of PD-L1^+^CECs exerted a beneficial influence on the response, we speculated that various PD-L1^+^CECs play disparate roles in the process of disease development. The reduction in multiploidy and the occurrence of other aneuploid PD-L1^+^ CECs indicate disease progression.

Case 169 was diagnosed with poorly differentiated gastric adenocarcinoma with multiple metastases (Fig. 5C). The Lauren histologic type was intestinal with HER2 IHC +  + status. Accordingly, this patient was treated with XELOX plus trastuzumab as first-line treatment; after seven cycles, the patient reached PD and continued to receive PTX plus trastuzumab and pembrolizumab as a second-line therapy (Fig. 5C-a). We monitored the dynamic enumeration and subtype variations in circulating PD-L1^+^ CTCs/CECs of the second-line therapy. Triploid-PD-L1^+^ CTCs were captured before the beginning of cycle two. Intra-therapeutic multiploid-PD-L1^+^ CECs were substituted with triploid-PD-L1^+^ CECs at PD (Fig. 5C-b). Accordingly, the tumor burden sharply progressed in 3 months (Fig. 5C-c-e). These results indicate that karyotype shifting following treatment is related to therapeutic resistance. Unlike multiploid PD-L1^+^ CECs, triploid and tetraploid PD-L1^+^ CECs contribute to acquired resistance.

## Discussion

Trastuzumab combined with the PD-1/PD-L1 checkpoint blockade immunotherapy has revolutionized the treatment of HER2-positive GC. Keynote-811 demonstrated that dual blockade of HER2 and PD-1 plus chemotherapy was effective as a first-line regimen in patients with HER2-positive GC as first-line regimen. To date, PD-L1 CPS and MSI status, but not HER2 status, are the only two factors that have been validated to be associated with clinical response. In the current study, we focused on the PD-L1 value for predicting clinical response and monitoring the disease process. Given the dynamic heterogeneity of gastric cancer following treatment, we applied SE-iFISH to explore PD-L1 expression and karyotypic features of CTCs and CECs following treatment and their relationship with therapeutic response.

The results indicated that the baseline PD-L1^+^ CTCs detected in HER2-negative patients were much lower than HER2-positive patients (0% vs. 14.75%, respectively). The relationship between PD-L1 and HER2 expression in tumor cells remains controversial, although some studies have reported that PD-L1 expression in GC is positively correlated with HER2 overexpression (84.6% vs. 51.6%) [21]. Most studies support the finding that PD-L1 expression occurs more frequently in the HER2-negative group than in HER2-positive cohorts [22]. Similarly, in the current study, the corresponding tissue PD-L1 positivity in HER2-negative and -positive patients was 33.3% (3/9) and 24.5% (15/61), respectively. Nevertheless, this was the opposite of PD-L1^+^ CTCs distribution in HER2-positive and -negative patients. In our study, there was no relationship between PD-L1 expression in tissue and CTCs, which was similar to the other reports [23–25]. The evasion into peripheral PD-L1^+^ tumor cells may also lead to the loss of PD-L1 in the tumor lesion.

Unlike the PD-L1 CPS, the existence of baseline PD-L1^+^ CTCs suggests that the patients did not benefit from anti-HER2 and anti-PD-1 therapy in this study. PD-L1^+^ CTCs have been widely investigated and are regarded as markers of poor prognosis in various tumors. However, whether this correlates with ICIs therapy outcomes remains unclear. Many studies have considered the presence of baseline PD-L1^+^ CTCs as a favorable factor in immunotherapy [24, 26]. It has also been reported that higher baseline PD-L1^+^ CTCs were observed in “non-responders” [27]. A systematic review and meta-analysis including 20 studies revealed that the expression of PD-L1 on CTCs was not related to the prognosis of patients treated with ICIs [28]. Extending beyond previous demonstrations of the relevance between baseline PD-L1^+^ CTCs quantity/positivity and prognosis, our study further analyzed ploidy patterns. The results indicate that triploidy and multiploidy accounted for a large proportion of baseline PD-L1^+^ CTCs. Triploid CTCs are believed to be involved in resistance to therapy and promotion of tumor progression [29, 30]. Thus, most baseline PD-L1^+^CTCs that escaped from tumor sites in our cohort exhibited a potent malignant phenotype, leading to therapy resistance. We therefore hypothesized that there was a CTC karyotype in the distribution heterogeneity in different cohorts that contributed to contrary conclusions. Although many studies regard PD-L1^+^ CTCs as a supplementary method to detect PD-L1 expression in tumors and use it as a reference for deciding the treatment regimen, our results proved the necessity of evaluating karyotypic features rather than quantity and phenotype alone. CECs and CTCs have disparate gene mutations, indicating their different roles in tumor progression [31]. The aneuploid CECs, which could be directly derived from malignant cancer cells, harbor mixed properties of both endothelial vascularization ability and cancerous malignancy in the tumor microenvironment. However, the clinical relevance of aneuploid CECs remains unclear. As described in our results, multiploid-PD-L1^+^ CECs that form after treatment contribute to a favorable response, whereas the main karyotype consisted of triploidy and tetraploidy at PD. Hence, the CEC karyotype changes dynamically following treatment, and triploid and tetraploid PD-L1^+^ CECs are associated with secondary resistance to dual HER2 and PD-1 blockade therapy. Proportion of PD-L1-positive endothelial cells also participate in the calculation of PD-L1 CPS, which could partially explain PD-L1 CPS failed to predict ICIs response. In this way, it is nowhere enough to evaluate PD-L1 CPS at baseline to screen for potential beneficiaries of anti-PD-1. The combination of dynamic karyotype detection and tumor tissue PD-L1 CPS could be more precise for predicting and monitoring therapeutic response. What’s more, anti-angiogenic therapy could be a potential treatment for patients with detected triploidy and tetraploidy PD-L1-positive CECs after treatment. Tumor endothelial cells (TECs) play an important role in vascular structure and physiology. As cells directly contact infiltrating immune cells, TECs expressing PD-L1 promote the suppression and apoptosis of tumor-infiltrating CD8^+^ T cells, resulting in tumor development [32]. We further classified PD-L1^+^ CECs based on their karyotypic features. The investigation of functions and contact with the immune microenvironment of different subtypes is warranted.

The mechanisms of dynamic alterations of karyotypes following immunotherapy or anti-HER2 plus anti-PD-1 treatment have not been explored. Even though it has also been reported that immune checkpoint inhibitors (ICIs) increased karyotype shifting in patients along with treatment [15], specific mechanisms have not been demonstrated. Certain researches revealed possible mechanisms. Alteration of karyotypes could be driven by endogenous and exogenous stressors. There is a high level of intra-tumoral DNA heterogeneity. It has been reported that with the process of tumor development, a shift in karyotypes can be explained by successive loss or gain of chromosomes combined with aberrant variation of genes involved in cell cycle regulation, resulting in cell populations with advantage of growth. [33] Treatment was also another factor leading to the karyotype shift. Agents that target the spindle apparatus, DNA replication, topoisomerases, hypoxia, proteasome, histone deacetylase, and cell cycle kinases were reported to participate in generating cells with aneuploid by increasing the risk of chromosomal instability (CIN) during mitosis [34]. CIN was considered as one of the most important factors in the generation of aneuploidy. During the process of treatment, tumor cells adapt CIN by obtaining specific aneuploid karyotype. Cells with novel karyotypes maintain a high level of fitness [35]. In this way, karyotype shift caused by treatment promoted tumor cells survival in new environment. What’s more, it has been reported that aneuploid cells could shape the microenvironment in which they and their daughter cells reside (the activation of immune microenvironment, etc.). Tumor microenvironment could also modulate the rate of generation of new karyotype and select for cells that are most fit under a new condition. Besides, researchers found that misexpression of activation induced cytidine deaminase (AID), induced by inflammation-mediated NF-κB signaling, can lead to DNA double-strand breaks, somatic mutations, and chromosomal aberrations. Hence, chronic inflammation and/or oxidative stress caused by treatment could have aneugenic effects on tumor cells, which lead to the shift of karyotypes [36].

## Conclusion

In conclusion, our research demonstrated that PD-L1^+^ CTCs/CECs exhibited diverse functions as indicators of therapy, and baseline PD-L1^+^ CTCs were associated with inferior prognosis, whereas post-treatment PD-L1^+^ CECs were associated with longer irPFS. Karyotypic features were also key factors in the development of therapeutic resistance, and the presence of triploid PD-L1^+^ CTCs at baseline facilitated disease progression, while multiploid-PD-L1^+^ CECs formed after treatment indicated a favorable prognosis, and triploid and tetraploid PD-L1^+^ CECs participated in acquired resistance. Our findings emphasize the clinical significance of the karyotypic features of PD-L1^+^ CTCs/CECs and their dynamic monitoring in therapeutic resistance. Limitations in this study included the small number of enrolled patients, the limited follow-up period, and the clinical treatment of the enrolled patients was not unified.

## Data Availability

The data generated or analyzed during this study are included in this published article and its supplementary information files, or are available from the corresponding author on reasonable request.
